# Usefulness of levels of 2-methylbutyrylglycine and 2-ethylhydracrylic acid in urine for diagnosing 2-methylbutyrylglycinuria

**DOI:** 10.1186/s13023-025-03865-3

**Published:** 2025-07-01

**Authors:** Hao Liu, Ming Xu, Min Chen, Yong Peng, Liang Ye, Yi-fan Yin, Jing-kun Miao

**Affiliations:** 1 Newborn Screening Center, Department of Pediatrics, Chongqing Health Center for Women and Children/Women and Children’s Hospital of Chongqing Medical University, 120 Longshan Road, Chongqing, 401147 China; 2 NHC Key Laboratory of Birth Defects and Reproductive Health, Chongqing Health Center for Women and Children/Women and Children’s Hospital of Chongqing Medical University, Chongqing, 401147 China

**Keywords:** Urine organic analysis, 2-methylbutyrylglycine, 2-methylbutyrylglycinuria, 2-ethylhydracrylic acid

## Abstract

**Background:**

The congenital disease 2-methylbutyrylglycinuria involves defective isoleucine metabolism and is currently diagnosed based on elevated levels of 2-methylbutyrylcarnitine/isovalerylcarnitine C5 in blood, followed by analysis of 2-methylbutyrylglycine (2-MBG) in urine at an early stage. However, 2-ethylhydracrylic acid (2-EHA) was reported as a more easily detected marker for 2-methylbutyrylglycinuria due to its larger volume in urine. Here we explored the usefulness of two urinary markers for diagnosing the condition.

**Methods:**

We used electrospray-tandem mass spectrometry to assay levels of C5 and other acylcarnitines in blood and gas chromatography-mass spectrometry to assay levels of 2-MBG and 2-EHA in the urine of 12 individuals diagnosed with 2-methylbutyrylglycinuria based on *ACADSB* genotyping and in 166 controls. The diagnostic sensitivity and specificity of 2-MBG and 2-EHA were assessed based on receiver operating characteristic curves. Potential associations of levels of the two urinary markers with levels of C5 and other acylcarnitines in blood were explored using Pearson correlation analysis.

**Results:**

Urine of the 12 individuals with 2-methylbutyrylglycinuria contained a 2-MBG level of 1.78–11.89 (reference range, 0–0) and a 2-EHA level of 37.80-373.13 (reference range, 0-28.69), but levels of both analytes were undetectable or barely detectable in the urine of the 166 controls. Both markers showed 100% diagnostic sensitivity, and 2-MBG showed slightly higher specificity than 2-EHA (2-MBA, 99.5%, 95%CI 0.970-1.00; 2-EHA, 97.8%, 95%CI 0.943–0.994). Levels of 2-MBG, but not 2-EHA, in urine showed a reliable Pearson correlation with levels of C5 in blood (R^2^ = 0.71, *P* = 0.0006), but not with the ratios of C5 to other acylcarnitines in blood.

**Conclusion:**

The urinary markers 2-MBG and 2-EHA show similar sensitivity for diagnosing 2-methylbutyrylglycinuria, but the lower specificity of 2-EHA and the fact that it can be elevated in several other congenital metabolic disorders means that it should be used as a diagnostic marker only in conjunction with other markers and clinical factors.

## Introduction

The rare, autosomal recessive disease 2-methylbutyrylglycinuria, also known as short/branched-chain acyl-CoA dehydrogenase deficiency (OMIM 610006), can manifest variously as delayed development, intellectual disability, seizures, muscular atrophy and/or hypotonia although many affected newborns may appear normal without obvious health problems [1–4]. Mutations in subunit B of acetyl-CoA dehydrogenase, encoded by the gene *ACADSB* (OMIM 600301), lead to insufficient activity of 2-methylbutyryl-CoA dehydrogenase, which metabolizes isoleucine as well as other branched amino acids [5]– [6]. This deficiency leads to accumulation of 2-methylbutyryl-CoA in the body, which in turn leads to accumulation of 2-methylbutyrylcarnitine/isovalerylcarnitine (C5) in the blood and 2-methylbutyrylglycine (2-MBG) in urine [7]. The disease 2-methylbutyrylglycinuria is currently diagnosed based on second-tier newborn screening for elevated C5 in blood, and it can be differentially diagnosed from isovaleric acidemia due to the elevated levels of 2-MBG in urine. Finally, the diagnosis can be confirmed through *ACADSB* genotyping [8]. This approach can fail to detect some cases because newborns may show only weakly elevated 2-MBG due to inadequate sensitivity of acylglycine and are therefore not subjected to genotyping [9].

Another potentially diagnostic marker of 2-methylbutyrylglycinuria may be 2-ethylhydracrylic acid (2-EHA), which is an intermediate in the alternative “R-pathway” of l-isoleucine metabolism and is easily detectable using gas chromatography-mass spectrometry available in most clinical laboratories [10–12]. Under normal conditions, the “S-pathway” of l-isoleucine metabolism predominates; but when this pathway is blocked, such as because of *ACADSB* mutations, the R-pathway increases, leading to higher levels of 2-EHA in urine [13]– [14].

Here we assessed the usefulness of urinary levels of 2-EHA and 2-MBG, as determined using gas chromatography-mass spectrometry, for diagnosing 2-methylbutyrylglycinuria.

## Methods

### Subjects

We retrospectively analyzed acylcarnitine results from 12 newborns who were definitively diagnosed with 2-methylbutyrylglycinuria at our medical center between January 2022 and December 2023 based on homo- or compound heterozygosity for known pathogenic or likely pathogenic mutations in *ACADSB*. The samples had been collected as part of routine newborn screening. We also retrospectively analyzed the results of organic acid analysis of urine from 166 individuals who came to our medical center during the same time period and whose C5 levels in blood were determined to be < 0.41 µmol/L and whom we therefore treated as controls in the present study. All individuals consented for their blood or urine to be collected and analyzed and for their anonymized medical data to be published for research purposes. This study was approved by the Ethics Committee at our hospital and all methods were performed in accordance with the relevant guidelines and regulations.

### Next-generation sequencing and analysis of genetic variants

Mutations in the *ACADSB* gene were determined using specific probe capture technology as follows. The target region was captured and sequenced using second-generation sequencing technology. The extracted genomic DNA that satisfied quality control criteria was homogenized and fragmented into pieces 100–500 bp long using the VAHTS Universal Plus Fragmentation Module (Novozyme). DNA fragments of 200–250 bp were separated using double selection based on magnetic beads, then adapters were added and the products were purified after another double selection. The purified products were amplified and again purified, then suspended in Tris-EDTA buffer. A DNA library was constructed, fragment concentrations were assayed, and fragments that satisfied quality control criteria were used for hybridization. The target region sequence was captured using a customized probe, and the hybridization library was pooled and quantified; the pooling library was then subjected to single-strand circularization and rolling circle replication. Next the circularized library was used to prepare DNA nanoballs, which were sequenced using a PE100 + 10 protocol on a high-throughput MGISEQ-2000 sequencer (MGI Tech Co., Ltd, Shenzhen, China).

### Biochemical analysis

Organic acids in urine were extracted using ethyl acetate, then the extract was derivatized using trimethylsilane and analyzed using gas chromatography-mass spectrometry on a QP2020 system (Shimadzu, Kyoto, Japan) in full scan mode. Following normalization to creatinine, urinary organic acid levels were semi-quantitatively determined as the ratio of the peak area of each target compound to that of the internal standard, expressed in arbitrary units. Levels of C5 and ratios of levels of C5 to free carnitine (C0), acetylcarnitine (C2) or propionylcarnitine (C3) in blood samples were determined using liquid-tandem mass spectrometry on an AB4500 system (Sciex, Framingham, MA, USA) and a NeoBase™ non-derivatized MSMS kit (PerkinElmer, Waltham, MA, USA).

### Statistical analysis

Microsoft Excel 2013 was used to analyze clinicodemographic data of subjects, while MedCalc -version 23.0.9 (MedCalc Software, Acacialaan, Ostend, Belgium) was used to assess diagnostic sensitivity and specificity based on receiver operating characteristic curves, as well as correlations of levels of 2-MBG and 2-EHA in urine with levels of C5 or the ratios of C5 to other acylcarnitines in blood based on Pearson correlation analysis.

## Results

**Fig. 1:**
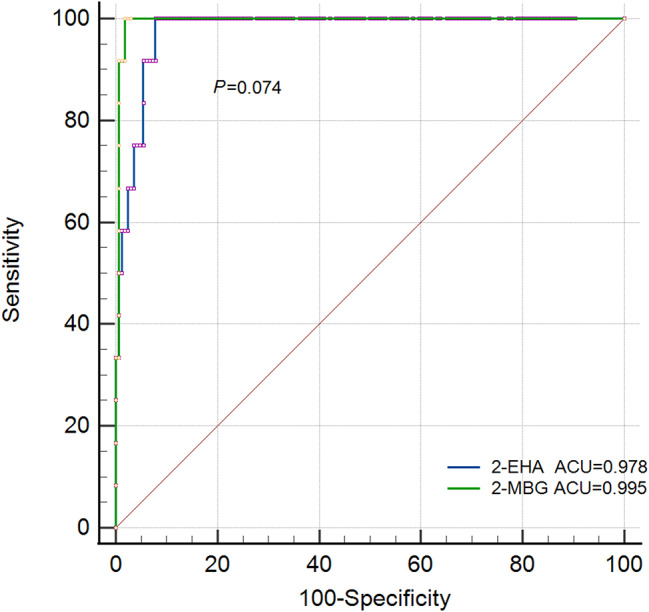
Ability of levels of 2-MBG or 2-EHA in urine to predict 2-methylbutyrylglycinuria in our total sample of 178 individuals. AUC, area under the receiver operating characteristic curve

In the 12 individuals diagnosed with 2-methylbutyrylglycinuria, levels of 2-MBG in urine ranged from 1.78 to 11.89, while levels of 2-EHA in urine ranged from 37.80 to 373.13 (Table 1). Both metabolites were well outside their normal reference ranges. Ten individuals showed elevated C5 in blood, ranging from 0.32 to 1.64 µmol/L; the remaining two individuals showed C5 levels within the normal reference range. In the total sample of 178 individuals (normal: patients, 166:12) level of 2-MBG in urine predicted disease with an area under the receiver characteristic curve (AUC) of 0.995 (95%CI 0.970-1.000), while level of 2-EHA in urine predicted it with an AUC of 0.978 (95%CI 0.943–0.994) (Fig. 1). The pairwise comparison of ROC was conducted with a P value of 0.074.

**Table 1:** Biochemical analysis of urine and blood of individuals with 2-methylbutyrylglycinuria

No.	Sex	Metabolites in urine		Acyl-carnitines in blood				Genotype ^c^
		2-MBG (0–0) ^a^	2-EHA (0-28.69)^a^	C5 (0.04–0.41)	C5/C0 (0-0.02)	C5/C2 (0-0.04)	C5/C3 (0-0.35)	
1	F	3.760	171.010	0.636	0.027	0.066	0.946	Com het
2	F	6.390	193.850	0.541	0.019^b^	0.062	0.811	Com het
3	M	2.800	40.250	0.548	0.017^b^	0.034^b^	0.346^b^	Com het
4	M	4.970	197.970	0.497	0.015^b^	0.036^b^	0.234^b^	Com het
5	M	8.440	118.050	1.010	0.022	0.083	0.835	Com het
6	M	1.780	22.310	0.776	0.018^b^	0.047	0.361	Com het
7	M	8.970	156.860	0.620	0.019^b^	0.033^b^	0.246^b^	Com het
8	M	7.410	373.130	0.860	0.037	0.075	1.000	Hom
9	M	8.430	90.160	1.010	0.022	0.083	0.835	Com het
10	F	2.850	37.800	0.386^b^	0.011^b^	0.021^b^	0.207^b^	Hom
11	M	3.370	63.200	0.320^b^	0.006^b^	0.013^b^	0.140^b^	Hom
12	F	11.890	97.920	1.640	0.063	0.170	1.220	Hom

Levels in blood are reported in µmol/L, while levels in urine are reported without units. F, female; M, male

Com het: compound heterozygotes; Hom, homozygous

a Normal reference range with 95% confidence interval

b Within the normal reference range

c Genotyping of the *ACADSB* gene

**Fig. 2:**
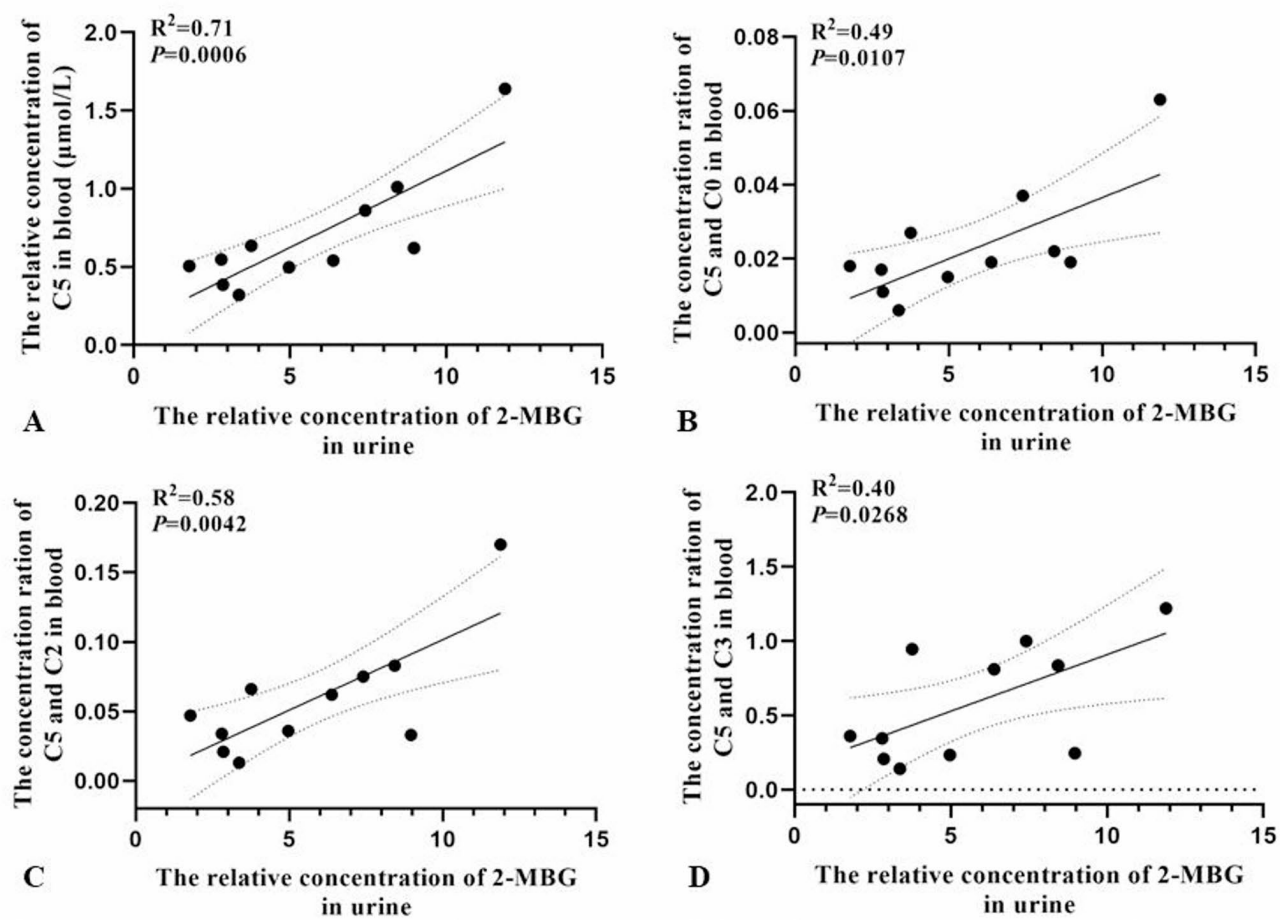
Pearson analysis of correlations of levels of 2-MBG in urine with levels of (**A**) C5 in blood or ratios of the levels of C5 to (**B**) C0, (C) C2 or (D) C3 in blood of individuals with 2-methylbutyrylglycinuria

**Fig. 3:**
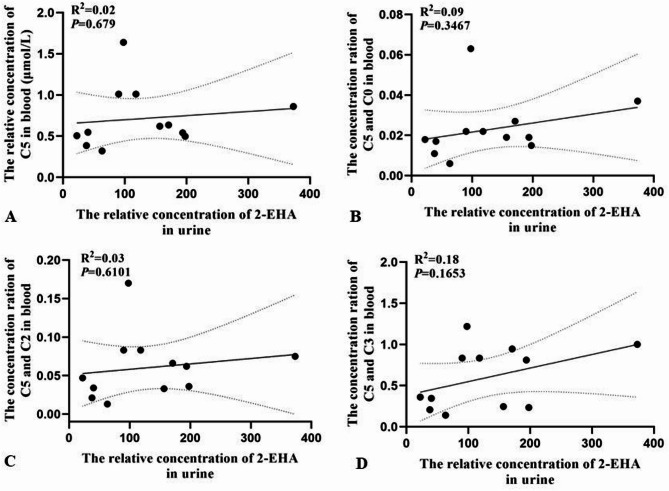
Pearson analysis of correlations of levels of 2-EHA in urine with levels of (**A**) C5 in blood or ratios of the levels of C5 to (**B**) C0, (C) C2 or (D) C3 in blood of individuals with 2-methylbutyrylglycinuria

**Fig. 4:**
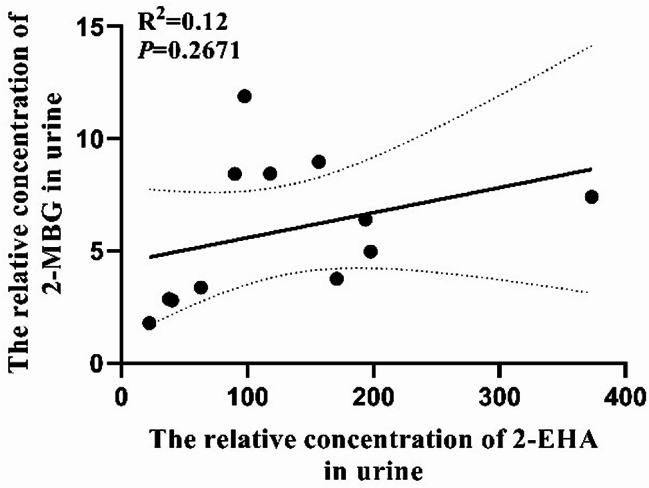
Analysis of potential correlation between levels of 2-MBG or 2-EHA in urine of individuals with 2-methylbutyrylglycinuria

Of the 12 individuals with 2-methylbutyrylglycinuria, seven showed elevated ratios of C5 to C3, six showed elevated ratios of C5 to C0, and five showed elevated ratios of C5 to C2 (Table 1). In these 12 individuals, levels of 2-MBG in urine correlated with levels of C5 in blood (R^2^ = 0.71, *P* = 0.001) (Fig. 2), but levels of 2-EHA in urine or ratios of levels of C5 to levels of other acylcarnitines in blood correlated negligibly with levels of C5 in blood (R^2^ < 0.600) (Fig. 3). Levels of 2-MBG and 2-EHA in urine did not correlate significantly with each other (Fig. 4).

## Discussion

Here we provide evidence that 2-MBG in urine can diagnose 2-methylbutyrylglycinuria with reasonable sensitivity and specificity, justifying its use in urine organic acid analysis for inborn error of metabolism as an ideal marker. While 2-EHA in urine shows similar sensitivity as 2-MBG, it shows lower specificity and is known to be elevated in several other inherited metabolic disorders such as methylmalonyl-CoA mutase deficiency, β-ketothiolase deficiency, and 2-methyl-3-hydroxybutyryl-CoA dehydrogenase deficiency which are defected in isoleucine at distal steps in the catabolic pathway [15–17]. In our study, 2-EHA was also found remarkably increased in urine of a patient with maple syrup urine disease which is a genetic disorder characterized by the defect of branched-chain amino acid catabolism. 2-EHA can origin from the distal defects of the S-pathway (such as β-ketothiolase deficiency), but also the proximal defects of the S-pathway as the case for 2-methylbutyrylglycinuria. In such case, 2-EHA is an intermediate in a minor R-pathway of isoleucine oxidation. S- and R-pathway can interconvert from each other by transaminase. A R-pathway intermediate is increased when the S-pathway is overloaded or interrupted. Therefore, it may be diagnostically useful only in conjunction with other markers or clinical factors due to the urinary excretion in multiple inborn errors of metabolism.

Our results are important for rational application of the current screening biomarkers, given that the level of C5 in blood is not a completely reliable marker. Indeed, two of our 12 individuals diagnosed with 2-methylbutyrylglycinuria showed a normal level of C5 in blood because the used upper limit of the reference (0.41 µmol/L) was a little higher than previously reported values (0.35 or 0.30µmol/L) [7, 18]. This implies that the upper limit of the normal reference range might need to be lowered in order to ensure no missed diagnosis, as suggested by other studies [19]– [20]. Each clinical laboratory may need to optimize its normal reference range to avoid missing cases during expanding newborn screening. Our results also suggest that ratios of C5 to other acylcarnitines in blood are not useful for differential diagnosis, in contrast to previous studies which showed that the absence of elevated C5 rations are highly suggestive of 2-methylbutyrylglycinuria instead of isovaleric acidemia [18, 21]. The discrepancy may need a larger sample analysis to explore intensively.

Our detection of 2-MBG in the urine of individuals with 2-methylbutyrylglycinuria but not in the urine of controls is consistent with a previous study [22]. We detected 2-EHA in the urine of controls, similar to a previous study [23], but it was present at much higher levels in the urine of individuals with 2-methylbutyrylglycinuria. Comparing with 2-MBG, 2-EHA is excreted more in urine of both healthy individuals and patients leading to perspective that 2-EHA is more easily detected as a biomarker. Nevertheless, while our analysis confirms that 2-methylbutyrylglycinuria involves excessive urinary excretion of 2-EHA, this analyte by itself is not sufficiently specific on its own to screen for the disease or diagnose it due to the urinary excretion of patients with some other inborn errors of metabolism. On the other side, 2-methylbutyrylglycinuria is considered as an asymptomatic disorder for about 90% of the identified patients and about 10% were reported as symptomatic [7]. In the Hmong population, the disorder is even likely benign. In the general population, the clinical course is poorly predictable. Increased 2-EHA in urine through the R-pathway was reported as a possible safe valve for over flow of accumulating S-pathway metabolites and therefore alleviate the severity of the disorder [10]. Along this line of thought, whether 2-EHA would be a potential marker to predict the prognosis of 2-methylbutyrylglycinuria is worth elaborately studying in the future.

Then, we explored the quantitative relationship between the markers in urine and C5 in blood or its ratios of the patients. It turned out that level of 2-MBG only correlated linearly with level of C5, which may reflect the fact that both are derived from increased 2-methylbutyryl-CoA due to deficiency of 2-methylbutyryl-CoA dehydrogenase. In contrast, level of 2-EHA did not correlate linearly with level of C5 or any ratio, which may reflect the fact that 2-EHA as an intermediate metabolite in R-pathway is not directly related metabolically to 2-methylbutyryl-CoA. There is no linear relationship between levels of 2-MBG and 2-EHA neither which is readily understandable due to the different metabolic pathways they were involved in. In general, urinary 2-MBG seems to be a more direct marker compared with 2-EHA, which is possibly predictable on the basis of C5 level in blood.

Biochemical markers in urine can diagnose 2-methylbutyrylglycinuria at an early stage. Compared with the most commonly used marker 2-MBG, 2-EHA offers similar sensitivity, but its specificity is slightly lower and is affected by more factors. It may therefore be inappropriate to use alone for diagnosing 2-methylbutyrylglycinuria. Further research should include more patients’ data for diagnostic test evaluation and explore whether the levels of 2-EHA are related to disease severity or prognosis for the reason that it is a metabolite of the compensatory R-pathway.

## Data Availability

All the data and materials will be made available upon request to the corresponding author.
